# Association between Time on Protease Inhibitors and the Incidence of Squamous Cell Carcinoma of the Anus among U.S. Male Veterans

**DOI:** 10.1371/journal.pone.0142966

**Published:** 2015-12-02

**Authors:** Pamela A. Mbang, Marc A. Kowalkowski, E. Susan Amirian, Thomas P. Giordano, Peter A. Richardson, Christine M. Hartman, Elizabeth Y. Chiao

**Affiliations:** 1 Department of Medicine, Baylor College of Medicine, Houston, TX, United States of America; 2 Houston Health Services Research and Development Center for Innovations in Quality, Effectiveness and Safety, Michael E. DeBakey Veterans Affairs Medical Center, Houston, TX, United States of America; 3 Department of Pediatrics, Baylor College of Medicine, Houston, TX, United States of America; 4 Dan L Duncan Cancer Center, Baylor College of Medicine, Houston, TX, United States of America; National Cancer Institute, UNITED STATES

## Abstract

Protease inhibitors (PIs) have been shown to have anti-tumor activity in addition to their antiretroviral properties. We sought to assess the association between PI use and the incidence of squamous cell carcinoma of the anus (SCCA) in HIV-infected individuals. We performed a retrospective cohort study among male US veterans diagnosed with HIV who were diagnosed between 1985 and 2010, using the Veterans Affairs HIV Clinical Case Registry (CCR). We calculated hazards ratios associated with PI use (both as percent time on PI and as 12-month intervals of PI use), utilizing time-dependent Cox models. We adjusted for risk factors, including age, race, year of enrolment into CCR, recent and nadir CD4, and percent time undetectable HIV viral load. A total of 28, 886 HIV-infected men met inclusion criteria. Of these, 373 were newly diagnosed with SCCA during the study period. In multivariate analysis, increasing percent time on PIs was associated with an increased risk of SCCA (aHR 1.07; 95% CI = 1.03–1.10 per 10% increase in time on PI). Poor immunologic recovery and virologic control, a history of condylomata acuminata, and CCR enrolment in the late combined antiretroviral therapy era were also associated with increased SCCA risk. Increasing percent time on a PI-based combined antiretroviral therapy regimen may be associated with an increased risk of developing SCCA in HIV-infected male US veterans. Future studies, better accounting for HIV control and treatment compliance, are necessary to further clarify this association.

## Introduction

HIV-infected individuals are at higher risk of developing cancer than the general population [1–4]. Although the introduction of combination antiretroviral therapy (cART) has led to an overall decline in AIDS-defining malignancies, such as Kaposi sarcoma and non-Hodgkin’s lymphoma, there have been increases in the incidence, morbidity, and mortality [1, 5–8] of squamous cell carcinoma of the anus (SCCA) and other non-AIDS-defining malignancies [7, 9, 10]. Among HIV-infected persons, men who have sex with men (MSM) [8, 11, 12] are at especially increased risk for SCCA, with the increase in SCCA incidence attributed by some researchers to the improved survival and aging of the HIV-infected population [4, 7]. Persistent immunodeficiency and persistent infection with oncogenic human papilloma viruses (HPVs) in the anal canal are thought to play a role in the pathogenesis of SCCA in HIV [13].

In addition to selectively binding to the catalytic site of HIV protease, interfering with HIV replication [14], HIV protease inhibitors (PIs), according to some limited evidence, may have antitumor properties [15, 16]. Some PIs (e.g., indinavir, saquinavir, ritonavir, lopinavir, and nelfinavir) at varying concentrations have been shown to be antiangiogenic and antitumorigenic because of their effects on cell invasion and matrix metalloproteinases, as well as because of modulation of the activity of cell proteasome [15–19]. They have, thus, been suggested as having potential therapeutic utility outside the context of HIV [20–22].

Given the general decline in AIDS-defining malignancies in the cART era and the anticancer effects attributed to the PIs *in vitro*, it seems counterintuitive that SCCA incidence is not declining. Thus, the objective of this study was to clarify the effect of PI use on the incidence of HIV-associated SCCA in male veterans registered in the Veteran Affairs (VA) HIV Clinical Case Registry (CCR), a nationwide registry containing health-related information on all known HIV-infected VA users in the United States [23, 24]. Additionally, we sought to evaluate other risk factors associated with HIV-associated SCCA in this population.

## Materials and Methods

This study was approved by the Institutional Review Board (IRB) of Baylor College of Medicine and the VA Research and Development Committee. Waiver of consent was obtained from the IRBs involved for this study. The VA registry from which data were extracted for this study includes thousands of patients from across the country, with limited protected health information and identifiers. The minor potential risk for loss of privacy through use of these data has been minimized, and the VA has considered this type of use of medical records data acceptable, as the risk to the individual is no more than minimal.

### Database

HIV-infected individuals were identified from the VA HIV clinical case registry (CCR). Established in 1992, the registry draws upon the electronic medical records of over 60,000 HIV-infected patients diagnosed after 1985 who were ever cared for by the VA. The VA HIV CCR includes demographic, laboratory, pharmacy, outpatient clinic visit, hospitalization data, International Classification of Diseases Ninth Revision (ICD-9) diagnostic codes from inpatient and outpatient encounters, and dates of death.

### Inclusion Criteria

**Fig 1:**
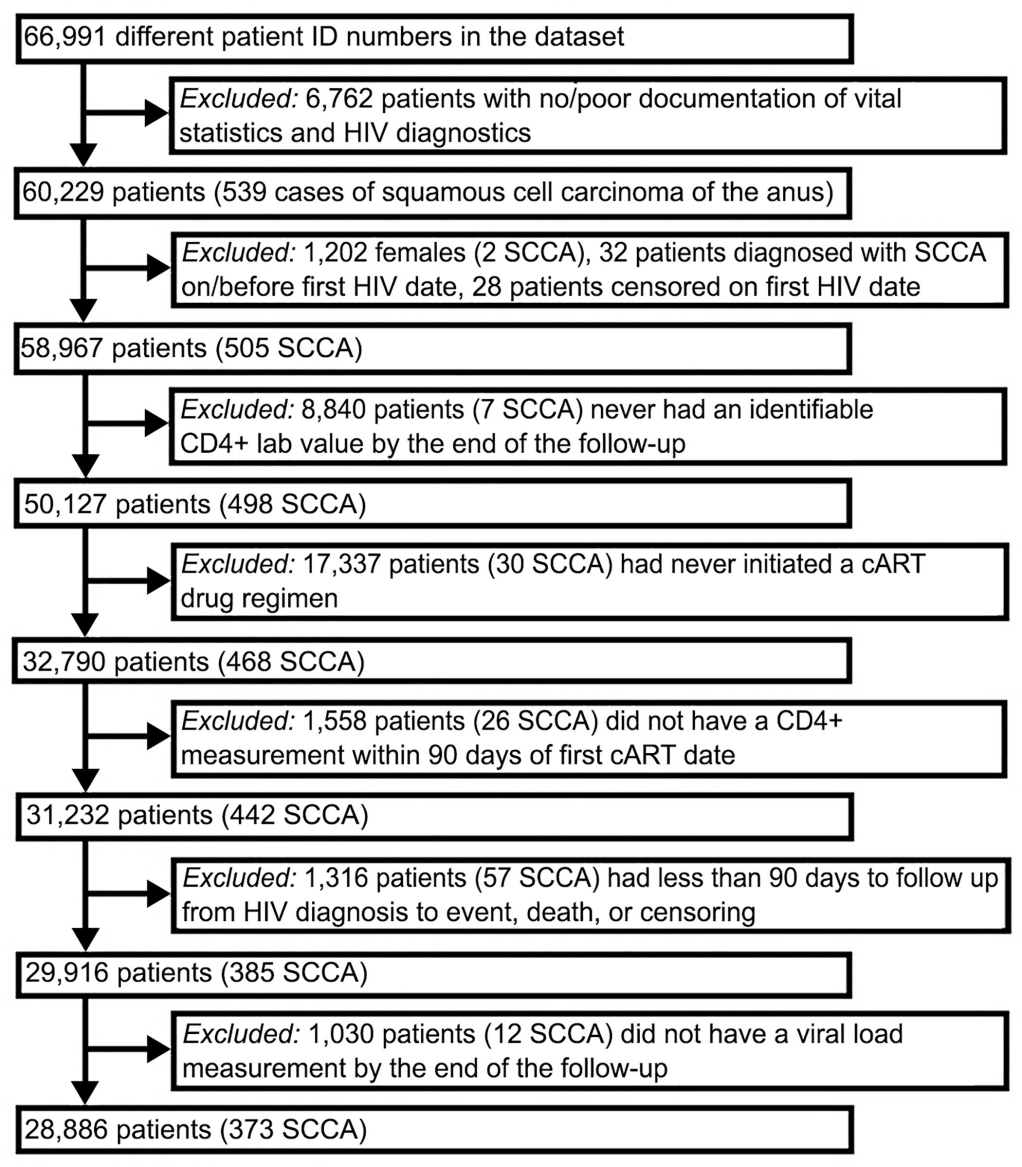
Flow Chart of Selected Criteria To Generate Final Cohort of HIV-infected Veterans.

The study population was restricted to HIV-infected veterans who were diagnosed between 1985–2010, were over the age of 18 with an identifiable HIV diagnosis date (based on the earliest ICD-9 code for HIV, the earliest positive HIV-related test, eg, ELISA, Western Blot, quantifiable HIV RNA measurement), or the earliest initiation of antiretroviral therapy (Fig 1). Individuals with only a single ICD-9 code for HIV and no lab or confirmatory pharmacy records were not included. Only men were included in our analyses because of the small number of HIV-infected women veterans (<2%). Additionally, we excluded individuals whose date of death or censor was the same as their initial HIV diagnosis date. We included only veterans ever receiving cART. Additionally, cART users with less than 90 days of follow-up or without quantifiable CD4 or HIV RNA measurements within 90 days of cART initiation were excluded.

### Primary Outcome

The primary endpoint was incident SCCA, identified from inpatient and outpatient ICD-9 codes 154.2 and 154.3. These SCCA ICD-9 codes have been previously validated in the VA and determined to have a positive predictive value of 88% [25]. Prevalent SCCA cases (i.e., individuals diagnosed before or within 90 days after the initial HIV diagnosis date) were excluded. The follow-up interval spanned from the index HIV diagnosis date to SCCA diagnosis, death, or December 31, 2010 (the final date of the current CCR iteration), whichever occurred first.

### Calculating Use of Combined Antiretroviral Therapy

cART use was abstracted from electronic pharmacy records available in the CCR database, which included prescriptions dispensed at all VA facilities. Use of cART among HIV-infected individuals was defined as any combination of 2 nucleoside reverse transcriptase inhibitors plus at least 1 non-nucleoside reverse transcriptase inhibitor (NNRTI), PI, integrase inhibitor, or CCR5 inhibitor; or any combination of 2 of the above classes of drugs. The duration of any cART and PI, NNRTI, and “other” cART regimens was estimated as the aggregate number of therapy days delivered from the dispensed prescriptions. The percent of total cART time each individual spent on PI, NNRTI, or “other” cART regimens was calculated as the duration of class-specific cART use divided by the duration of total cART use. Twelve months increments of PI use were also examined in a *post-hoc* analysis.

### Covariate Definitions

Potential confounders included patient age at HIV diagnosis and race/ethnicity, comorbid conditions as assessed by the Deyo modification of the Charlson comorbidity index (excluding points allotted for diagnosis of HIV infection) [26, 27], and the era of HIV diagnosis (pre-cART <1996, early cART 1996–2001, late cART 2002–2010). Additional HIV disease factors were captured from the CCR laboratory database. Specifically, pretreatment immune function was estimated from the nadir CD4 count prior to cART initiation. Percent of time of follow-up time on CART was defined as the total amount of time the subject was treated with CART divided by their total follow-up time. CD4 and HIV RNA measurements were also collected throughout the follow-up interval to monitor the effect of fluctuations in immune status throughout the follow-up period. CD4 variables were categorized as <200, 200–350, and >350 cells/μL. Nadir CD4 (or pre-treatment CD4) was measured prior to initiation of cART, and most recent CD4 was measured as at the time of event or censor. We used the number of HIV viral-load measurements per patient per year to adjust for frequency of HIV viral-load evaluations of patients in the cohort. HIV RNA was modeled as the percent of time undetectable and categorized as <40%, 40–79%, and ≥80%. Due to different laboratory assays in use at the VA facilities over all study years, the value for undetectable HIV RNA was established as <500 copies/mL.

### Statistical Analysis

All analyses were performed using SAS^®^ version 9.1 (SAS Institute, Cary, NC). The distributions of patient characteristics and cART use among the study cohort were calculated in the overall study cohort and stratified by SCCA status. The distributions of time-varying factors (e.g., recent CD4 and percent time with undetectable HIV viral load) were captured at the last follow-up. Additionally, we computed the crude incidence rate of SCCA by dividing the number of SCCA cases by person-years of follow-up.

Time-dependent Cox regression models were utilized to evaluate the effect of use of each class of cART on the risk of SCCA. To control for potential confounding, multivariable time-dependent Cox regression models were fit and adjusted for clinically relevant covariates described above (i.e., age at HIV diagnosis, race/ethnicity, illicit drug use, era of HIV diagnosis, Deyo comorbidity score, nadir and recent CD4, percent of time with undetectable HIV RNA, and number of HIV RNA measurements per year). Sensitivity analysis was conducted to verify study findings in separate, restricted cohorts, excluding individuals who were diagnosed with HIV before the introduction of cART in 1996 to account for the different treatments available to infected individuals during these time intervals.

## Results

Fig 1 describes the 66,991 HIV-positive veterans who were identified in the registry between 1985–2010.

### Study Cohort

A total of 28,886 HIV-infected male veterans (43%) met inclusion criteria. Of these, 373 were newly diagnosed with SCCA during the study period, giving a incidence rate of 1.38 per 1000 person-years. Individuals who were exposed to PIs had a longer median follow-up time than those who never took PIs (S1 Table).

### Patient Characteristics

Table 1 describes the cohort and presents univariate analyses of variables associated with SCCA. The median age at enrolment into the registry was 44 years. Racial minorities constituted over half of this study population. Just under one fifth of the patients had a history of anal condylomata acuminata. The number of subjects enrolled into the registry was evenly distributed across the pre-, early-, and late-cART eras. Most patients in the cohort had been exposed to a PI or to an NNRTI; and a much smaller percentage had been exposed to other cART, such as Integrase Inhibitors and CCR5 Inhibitors. Individuals without SCCA completed a median of 2.9 HIV viral load tests per year (quartile range: 2.24), whereas individuals who were diagnosed with SCCA completed significantly more tests per year on average (median: 4.3; quartile range: 4.50; p from Kruskal-Wallis test < .0001).

**Table 1:** Population Characteristics, Overall and by Squamous Cell Carcinoma of the Anus.

	*Overall (N = 28*,*886)*	*No SCCA (N = 28*,*513)*	*SCCA (N = 373)*
	*Median (IQR)*	*Median (IQR)*	*Median (IQR)*
**Age at HIV diagnosis (per year)**	44 (37–51)	44 (37–51)	42 (35–49)
% Time on cART (continuous)	56 (27–86)	56 (27–86)	52 (32–80)
% CART time on PI (continuous)	74 (2–100)	74 (2–100)	86 (64–100)
% CART time on NNRTI (continuous)	32 (0–95)	32 (0–95)	22 (0–58)
% CART time on Other cART (continuous)	0 (0–0)	0 (0–0)	0 (0–0)
	*N (%)*	*N (%)*	*N (%)*
**Type of cART utilization**			
PI ever	21782 (75)	21452 (75)	330 (88)
NNRTI ever	21153 (73)	20885 (73)	268 (72)
Other cART ever	3288 (11)	3253 (11)	35 (9)
**Race/ethnicity**			
White	10906 (38)	10681 (37)	225 (60)
Black	14479 (50)	14355 (50)	124 (33)
Hispanic	2219 (8)	2204 (8)	15 (4)
Unknown/ Other	1282 (4)	1273 (5)	9 (3)
**Year of HIV Diagnosis**			
Pre-cART era 1985–1995	8710 (30)	8532 (30)	178 (48)
Early cART era 1996–2001	10113 (35)	9985 (35)	128 (34)
Late cART era 2002–2010	10063 (35)	9996 (35)	67 (18)
**Illicit Drug Use**			
No	18667 (65)	18380 (64)	287 (77)
Yes	10219 (35)	10133 (36)	86 (23)
**Deyo Score without AIDS**			
0	15033 (52)	14818 (52)	215 (58)
1	8551 (30)	8438 (30)	113 (30)
2 and above	5302 (18)	5257 (18)	45 (12)
**Anal Condylomata Acuminata**			
No	23349 (81)	23232 (81)	117 (31)
Yes	5537 (19)	5281 (19)	256 (69)
**Nadir CD4 count prior to cART**			
CD4 > 350	6805 (24)	6756 (24)	49 (13)
CD4 200–350	7061 (24)	6984 (24)	77 (21)
CD4 < 200	15020 (52)	14773 (52)	247 (66)
**Recent CD4 count (cells/mm** ^**3**^ **)**			
CD4 > 350	15973 (56)	15820 (56)	153 (41)
CD4 200–350	5324 (18)	5223 (19)	101 (27)
CD4 < 200	7331 (26)	7213 (26)	118 (32)
**% time undetectable HIV RNA**			
<40%	12086 (42)	11846 (42)	240 (64)
40–80%	9301 (32)	9216 (32)	85 (23)
>80%	7499 (26)	7451 (26)	48 (13)

CCR = Clinical Case Registry; cART = combined antiretroviral therapy; NNRTI = non-nucleoside reverse transcriptase inhibitor; PI = protease inhibitor; SCCA = squamous cell carcinoma of the anus.

### Factors Associated with the Risk of SCCA

In the univariate analyses, Blacks and Hispanics had a lower risk of SCCA (HR = 0.42; 95%CI = 0.34–0.52 and HR = 0.31; 95%CI = 0.18–0.52, respectively), than Whites. A history of anal condylomata acuminata was associated with increased risk of SCCA (HR = 6.85; 95%CI = 5.50–8.53). In addition, patients who were enrolled in the late cART era as compared with the pre-cART era had an increased risk of SCCA (HR = 2.08; 95%CI = 1.49–2.91).

Regarding cART use, a longer relative time on cART (HR = 1.04 per 10% increase in percent follow-up time on cART; 95%CI = 1.01–1.08) and, specifically, a higher percent of CART time on a PI (HR = 1.09 per 10% increase in percent follow-up time on PI; 95%CI = 1.06–1.12) were associated with increased risk of SCCA. On the other hand, a longer relative percent of CART time on an NNRTI or other non-PI-based regimen was associated with decreased risk (HR = 0.95; 95%CI = 0.93–0.98 and HR = 0.85; 95%CI = 0.73–0.99, respectively).

CD4 and HIV RNA loads were also associated with the risk of SCCA development. Individuals with a CD4 nadir prior to cART or a recent CD4 ≤350 were at increased risk for SCCA. Furthermore, patients whose HIV viral loads were undetectable for 40–80% (HR = 0.44; 95%CI = 0.34–0.56) and >80% (HR = 0.56; 95%CI = 0.41–0.77) of the follow-up time were less likely to develop SCCA than were patients with less viral suppression.

**Fig 2:**
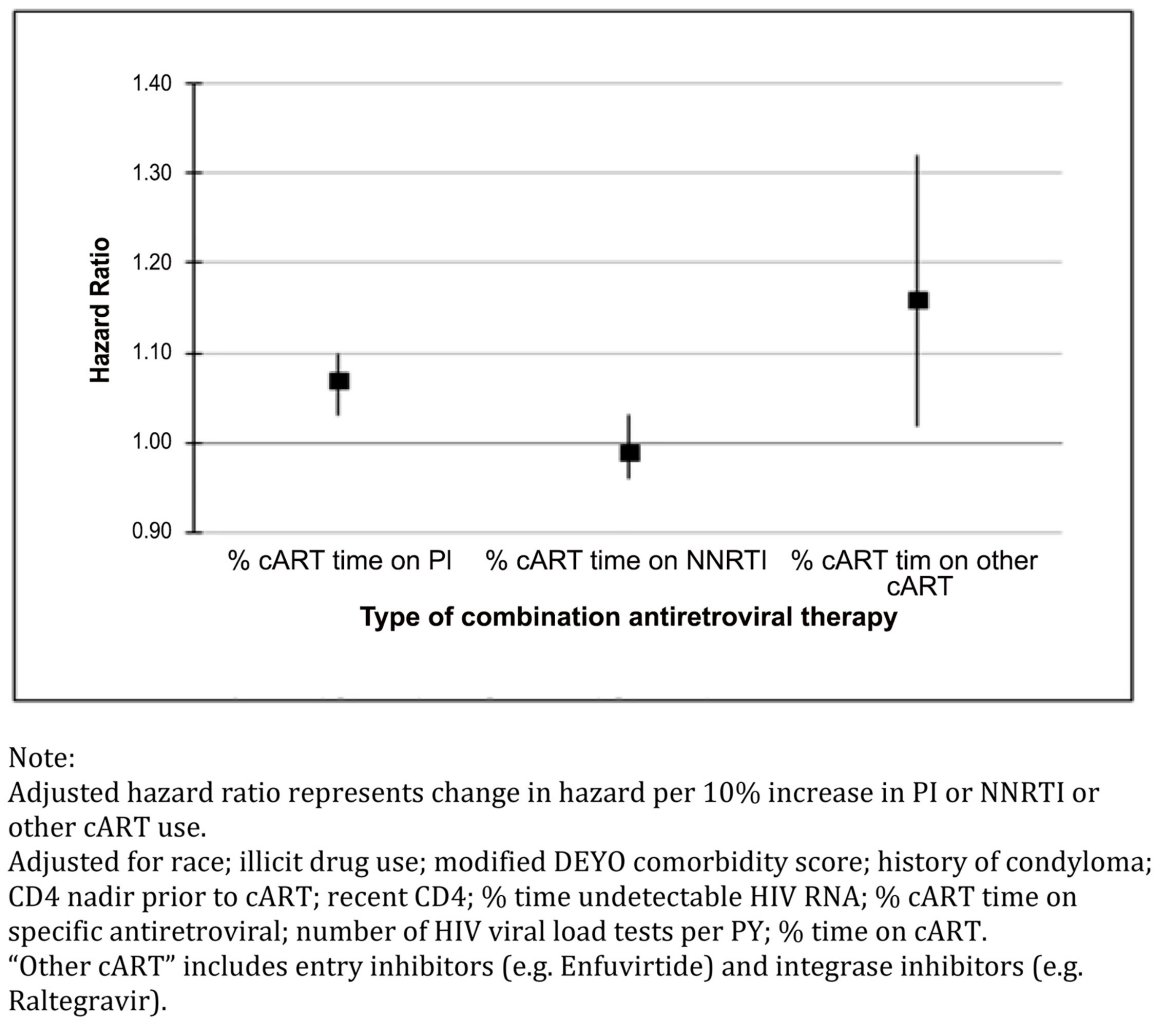
Box and Whisker Plot Showing Squamous Cell Cancer of the Anus Risk Associated with Protease Inhibitor, Non-Nucleoside Reverse Transcriptase Inhibitor, and Other CART Use.

Results from the multivariable regression analysis evaluating the effect of PI use on the risk of SCCA (adjusted for race, illicit drug use, comorbidity score, cART era, condyloma, CD4 nadir prior to cART, recent CD4, percent undetectable HIV RNA, number of HIV measurements per year, and time on cART) showed that increasing percent time on a PI was associated with a statistically significant increase in the risk of developing SCCA (adjusted hazard ratio per 10% increase, aHR = 1.07; 95%CI = 1.03–1.10; Fig 2). Table 2 provides the results from this analysis in tabular form. Similarly, the percent time on “other” (non-PI, non-NNRTI based) cART was also associated with an increased risk of developing SCCA (aHR = 1.16; 95%CI = 1.02–1.32). The relationship between NNRTI use and SCCA risk was not significant (Table 3). Results from sensitivity analyses of patients who were enrolled in the registry during the late cART era were similar to the results including the entire cohort (data not shown). In addition, stratification of multivariable models by history of condylomata acuminata provided no evidence of effect modification by this factor.

**Table 2:** Regression Analysis–Association between Protease Inhibitors (with Percent Time on cART and Percent Time Undetectable HIV RNA) & Time to Development of Squamous Cell Carcinoma of the Anus.

	Number of SCCA	Total Person-Years	Crude HR (95% CI)	Adjusted HR (95% CI)	P for adjHR
**Age at HIV Diagnosis** (per year)	-	-	1.01 (1.00–1.02)	1.01 (1.00–1.03)	0.0368
**Race**					
White	225	102970	1	1	
Black	124	135570	0.42 (0.34–0.52)	0.56 (0.44–0.72)	< .0001
Hispanic	15	21839	0.31 (0.18–0.52)	0.38 (0.22–0.64)	0.0003
Unknown	9	7848	0.61 (0.32–1.20)	0.73 (0.37–1.43)	0.3548
**Illicit Drug Use**					
No	287	159381	1	1	
Yes	86	108847	0.40 (0.32–0.51)	0.86 (0.66–1.13)	0.2863
**Year of HIV Diagnosis**					
Pre-cART era 1985–1995	178	123893	1	1	
Early cART era 1996–2001	128	97961	1.24 (0.96–1.59)	1.03 (0.77–1.38)	0.8266
Late cART era 2002–2010	67	46373	2.08 (1.49–2.91)	2.03 (1.34–3.07)	0.0009
**DEYO**					
0	215	188112	1	1	
1	113	56683	0.66 (0.53–0.83)	1.10 (0.86–1.40)	0.4554
2+	45	22074	0.44 (0.32–0.61)	1.11 (0.79–1.55)	0.5561
**Anal Condylomata Acuminata**					
No	117	205711	1	1	
Yes	256	62517	6.85 (5.50–8.53)	5.44 (4.31–6.87)	< .0001
**CD4 nadir prior to cART**					
>350	104	66627	1	1	
200–350	73	70612	1.47 (1.03–2.10)	1.36 (0.94–1.97)	0.1000
<200	196	129629	2.75 (2.02–3.73)	1.82 (1.30–2.54)	0.0005
**Recent CD4** (cells/mm^3^)					
>350	151	141679	1	1	
200–350	102	58574	2.13 (1.66–2.74)	1.29 (0.99–1.68)	0.0629
<200	120	53845	2.21 (1.73–2.81)	1.38 (1.04–1.82)	0.0255
**% time undetectable HIV RNA**					
>80%	48	39013	0.44 (0.34–0.56)	0.56 (0.38–0.81)	0.0025
40–80%	85	55779	0.56 (0.41–0.77)	0.52 (0.39–0.69)	< .0001
<40%	240	172078	1	1	
**% CART time on PI** (per 10%)	-	-	1.09 (1.06–1.12)	1.07 (1.03–1.10)	< .0001
**Number of HIV viral loads per PY**	-	-	1.19 (1.16–1.21)	1.18 (1.16–1.21)	< .0001
**% time on cART** (per 10%)	-	-	1.04 (1.01–1.08)	1.03 (0.99–1.07)	0.2249

CCR = Clinical Case Registry; cART = combined antiretroviral therapy; PI = protease inhibitor; PY = person per year; SCCA = squamous cell carcinoma of the anus.

**Table 3:** Regression Analysis–Association between NNRTI (with Percent Time on cART and Percent Time Undetectable HIV RNA) & Time to Development of Squamous Cell Carcinoma of the Anus.

	Adjusted HR (95% CI)	p
**Age at HIV Diagnosis** (per year)	1.01 (1.00–1.03)	0.0407
**Race**		
White	1	
Black	0.56 (0.44–0.72)	< .0001
Hispanic	0.38 (0.22–0.64)	0.0003
Unknown	0.74 (0.38–1.45)	0.3741
**Illicit Drug Use**		
No	1	
Yes	0.88 (0.67–1.15)	0.3320
**Year of HIV Diagnosis**		
Pre-cART era 1985–1995	1	
Early cART era 1996–2001	0.97 (0.72–1.29)	0.8109
Late cART era 2002–2010	1.75 (1.15–2.64)	0.0085
**DEYO**		
0	1	
1	1.09 (0.85–1.39)	0.4958
2+	1.10 (0.79–1.54)	0.5727
**Anal Condylomata Acuminata**		
No	1	
Yes	5.46 (4.33–6.89)	< .0001
**CD4 nadir prior to cART**		
>350	1	
200–350	1.37 (0.95–1.98)	0.0972
<200	1.86 (1.33–2.60)	0.0003
**Recent CD4** (cells/mm^3^)		
>350	1	
200–350	1.32 (1.01–1.72)	0.0428
<200	1.46 (1.10–1.93)	0.0082
**% time undetectable HIV RNA**		
>80%	0.50 (0.34–0.73)	0.0004
40–80%	0.51 (0.38–0.68)	< .0001
<40%	1	
**% cART time on NNRTI** (per 10%)	0.99 (0.96–1.03)	0.7047
**Number of HIV viral loads per PY**	1.18 (1.16–1.21)	< .0001
**% time on cART** (per 10%)	1.05 (1.01–1.09)	0.0233

CCR = Clinical Case Registry; cART = combined antiretroviral therapy; NNRTI = non-nucleoside reverse transcriptase inhibitors

Note. Event counts, total person-time, and crude hazard ratios for each variable provided in Table 2.

In order to allow for easier interpretability of our results, we conducted a set of *post-hoc* analyses in which we examined the effects of 12-month intervals of PI use on SCCA risk, using multivariable time-dependent modeling (data not shown). Being on PIs for 12–23 months was associated with 1.53 times (95% CI = 1.06–2.20) the SCCA risk of being on PIs for <12 months, adjusting for relative time on cART, race/ethnicity, illicit drug use, Deyo score, HIV diagnosis era, presence of condyloma, nadir and recent CD4 counts, percent time undetectable HIV RNA, and number of HIV RNA tests. Furthermore, being on PIs for 24–35 months was associated with almost twice the SCCA risk compared to being on PIs for <12 months (aHR = 1.97; 95% CI = 1.33–2.93), as was being on PIs for 36+ months (aHR = 1.97; 95% CI = 1.32–2.93).

## Discussion

In this nationwide, retrospective cohort study of HIV-positive US male veterans over a 25-year period, our findings did not suggest that the use of PIs lowered the incidence of SCCA in HIV patients; rather, there was a slight increase of SCCA risk associated with higher PI utilization.

In *in vitro* studies, PIs have been shown to target several pathways that play a role in the pathogenesis of various cancers. Nelfinavir targets the phosphoinositide 3-kinase/AKT pathway, thought to play an important role in the development of cancers through multiple mechanisms [28, 29]. In cervical carcinoma, saquinavir and ritonavir were shown to inhibit CIN cell invasion by their action on matrix metalloproteinases 2 and 9 [18]. There are no in vitro data available regarding the pathways targeted by specific PIs in SCCA, but SCCA closely resembles cervical cancer in several ways [30, 31]. They both have the same virologic etiology [32, 33], as well as similar precursor lesions, anal intraepithelial neoplasia [34] and cervical intraepithelial neoplasia, both of which may progress to SCCA and cervical cancer, respectively.

Contrary to the in vitro data, the current analysis and previous similar analyses show that despite adjusting for multiple variables, including percent of time with undetectable HIV viral load, current and nadir CD4, and the percent of follow-up time on CART, a greater percent of cART time on PI was associated with an increased risk of developing SCCA. Chao et al. [35] reported a similar finding in their study to evaluate the effect of cART use on cancer risk. In a cohort study of 12,872 HIV-positive individuals, an increased risk of anal cancer (but not any other non-AIDS-defining malignancy) was associated with increasing duration of PI utilization (HR = 1.16 per 1-year intervals; 95%CI = 1.02–1.31). Our study builds on that previous study, which did not adjust for overall relative time on cART and is in a larger cohort.

Although the biologic mechanism of our observation is unknown, an alternative explanation for our findins is that the use of PIs often in salvage cART regimens may be another indicator (above and beyond the cumulative HIV viral measure) of poor prior cART compliance and virologic control. This hypothesis is further supported by the fact that individuals with a higher percent time on “other cART” medications (ie, Integrase and Entry Inhibitors) were also at higher risk for SCCA. Most of these medications were also used in salvage cART regimens during the time period of this study. Additionally, it is also possible that we did not completely account for all confounders, including sexual orientation, which could confound the results if MSM are more likely to have longer PI utilization, although there are no currently published data indicating a relationship between PI-usage specifically and MSM.

We also found that SCCA risk was increased in patients diagnosed with HIV during the late cART era. This finding is somewhat consistent with studies that have shown an increase in the incidence of SCCA in HIV-infected persons since the introduction of cART [1, 2, 10, 12, 36–40]. This increase, as well as the increase in the risk of other NADMs after the introduction of cART [7, 9, 10], is likely because of improved HIV control and decreased competing morbidity and mortality associated with HIV and its opportunistic infections. In addition, similar to previous reports, this multivariate analysis confirmed that nadir CD4 count prior to initiation of cART and recent CD4 and HIV viral load were significant risk factors associated with the development of SCCA. These results suggest that the immunosuppression related to HIV may directly contribute to the development of anal cancer [8, 11, 37, 41, 42], possibly by impairing the cell-mediated immune control of HPV [43]. Previous prospective and cross-sectional studies have shown that a low CD4 is associated with an increased risk for AIN 2 or 3 [44–47]. In addition, Van der Snoek et al. [48] found that the use of cART was associated with a lower prevalence of HPV and AIN in a cross-sectional study of 250 HIV-infected MSM. Thus, taken together, previous studies suggest that initiating cART at higher CD4 counts and improved HIV viral control associated with cART utilization do appear to decrease SCCA risk, but that specific cART medications, specifically PIs, in and of themselves, may not provide any direct effect above and beyond HIV virologic control.

The Veterans Health Administration is the largest integrated healthcare system and provider of comprehensive HIV care in the United States [23]. To the best of our knowledge, this is the largest study published to date evaluating the association between the use of PI and the risk of developing HIV-associated SCCA. The main strength of this study is the study population; our large sample size allowed us to assess the risk of SCCA nationwide over the entire course of the HIV epidemic, despite the rarity of this malignancy. Furthermore, our analyses supersede previous comparisons of PI utilization centered on use versus no use by exploring the potential dose-response effect by using the percent cART time on PI. Thus, the analyses adjusted for both the duration of use and changes to the treatment regimen.

Our study also has several limitations. First, it is a retrospective study and, thus, may be subject to unmeasured confounders and/or residual confounding. However, there are no prospective, randomized controlled trials to evaluate the impact of PIs on the risk of developing SCCA; and such a study would be prohibitively large, given the rarity of SCCA. Second, our diagnoses were based on ICD-9 codes, which may not always be accurate. However, the anal cancer ICD-9 codes 154.2 and 154.3 used in the VA healthcare system have been previously validated and determined to have a positive predictive value of 88% [25]. Third, we do not have a direct measurement for categorizing MSM. MSM are at an increased risk for developing SCCA [8, 11, 12], likely because they have much higher rates of high-risk HPV infection. However, we did adjust for presence of condylomata acuminata (a variable that was highly associated with SCCA), which may have been a surrogate marker for high-risk HPV infection. Fourth, we did not evaluate anal-cancer screening procedures; however, there are currently no national VA anal-cancer screening guidelines, and no national practice of anal-cancer screening. Finally, we conducted our study among male veterans; thus, the generalizability of these data to other populations may be limited. Additionally, as a large number of individuals had to be excluded from our study (Fig 1), selection bias may have impacted our findings. However, based on the information available to us, we are unable to determine how different the excluded individuals were from our study population.

## Conclusion

Increasing percent time on a PI-based cART regimen was associated with increased risk of developing SCCA in HIV-infected male US veterans. This association may be a reflection of unmeasured risk factors, such as cART compliance and HIV control. At the very least, these data refute in vitro data suggesting a protective effect of PIs on HPV-associated anal cancer. Further studies are necessary to determine whether there are direct effects of PIs on HPV infection or the incidence of anal dysplasia.

## Supporting Information

S1 Table Median follow-up times in years for participants with and without squamous cell carcinoma of the anus by combination antiretroviral therapy (cART) use. (DOCX)
