# Supplementary material for: Association between Time on Protease Inhibitors and the Incidence of Squamous Cell Carcinoma of the Anus among U.S. Male Veterans
Source: PLoS One. 2015 Dec 2;10(12):e0142966. doi: 10.1371/journal.pone.0142966 (PMC4668039; doi:10.1371/journal.pone.0142966)
Supplement: S1 Table — (DOCX) [file pone.0142966.s001.docx]

|  |  | **No SCCA** | **SCCA** |
| --- | --- | --- | --- |
|  |  | Median Follow-Up Time  *Years (SD)* | Median Follow-Up Time  *Years (SD)* |
| **PI ever** | Yes | 9.9 (5.4) | 8.2 (4.8) |
|  | No | 5.9 (4.7) | 3.7 (4.7) |
| **NNRTI ever** | Yes | 9.5 (5.4) | 8.4 (4.8) |
|  | No | 6.6 (5.2) | 6.1 (5.0) |
| **Other cART ever** | Yes | 12.4 (5.6) | 13.7 (5.2) |
|  | No | 8.3 (5.3) | 7.5 (4.8) |

SD=standard deviation

**S1 Table.**  Median follow-up times in years for participants with and without squamous cell carcinoma of the anus by combination antiretroviral therapy (cART) use
